# The Adrenal Cortex, an Underestimated Site of SARS-CoV-2 Infection

**DOI:** 10.3389/fendo.2020.593179

**Published:** 2021-01-08

**Authors:** Yanfei Mao, Bo Xu, Wenbin Guan, Dunfeng Xu, Feng Li, Rongrong Ren, Xiaoyan Zhu, Yuan Gao, Lai Jiang

**Affiliations:** ^1^ Department of Anesthesiology and Surgical Intensive Care Unit, Xinhua Hospital, Shanghai Jiaotong University School of Medicine, Shanghai, China; ^2^ Department of Pathology, Xinhua Hospital, Shanghai Jiaotong University School of Medicine, Shanghai, China; ^3^ Department of Respiratory and Critical Care Medicine, Shanghai Public Health Clinical Center Affiliated to Fudan University, Shanghai, China; ^4^ Department of Physiology, Navy Medical University, Shanghai, China; ^5^ Department of Critical Care Medicine, Renji Hospital, Shanghai Jiaotong University School of Medicine, Shanghai, China

**Keywords:** severe acute respiratory syndrome coronavirus 2, coronavirus disease 2019, critically ill patients, adrenal cortex, adrenal insufficiency

## Abstract

**Background:**

The majority of the critically ill patients may have critical illness-related corticosteroid insufficiency (CIRCI). The therapeutic effect of dexamethasone may be related to its ability to improve cortical function. Recent study showed that dexamethasone can reduce COVID-19 deaths by up to one third in critically ill patients. The aim of this article is to investigate whether SARS-CoV-2 can attack the adrenal cortex to aggravate the relative adrenal insufficiency.

**Methods:**

We summarized the clinical features of COVID-19 reported in currently available observational studies. ACE2 and TMPRSS2 expression was examined in human adrenal glands by immunohistochemical staining. We retrospectively analyzed serum cortisol levels in critically ill patients with or without COVID-19.

**Results:**

High percentage of critically ill patients with SARS-COV-2 infection in the study were treated with vasopressors. ACE2 receptor and TMPRSS2 serine protease were colocalized in adrenocortical cells in zona fasciculata and zona reticularis. We collected plasma cortisol concentrations in nine critically ill patients with COVID-19. The cortisol levels of critically ill patients with COVID-19 were lower than those in non-COVID-19 critically ill group. Six of the nine COVID-19 critically ill patients had random plasma cortisol concentrations below 10 µg/dl, which met the criteria for the diagnosis of CIRCI.

**Conclusion:**

We demonstrate that ACE2 and TMPRSS2 are colocalized in adrenocortical cells, and that the cortisol levels are lower in critically ill patients with COVID-19 as compared to those of non-COVID-19 critically ill patients. Based on our findings, we recommend measuring plasma cortisol level to guide hormonal therapy.

## Introduction

In December 2019, Wuhan, the capital of Hubei province in China, became the centre of an outbreak of severe respiratory illness caused by a novel coronavirus, severe acute respiratory syndrome coronavirus 2 (SARS-CoV-2), which was later designated coronavirus disease 2019 (COVID-19) by WHO (1). COVID-19 that has a high sequence similarity (~80%) with SARS-CoV, triggered a global pandemic of novel COVID-19-infected pneumonia (NCIP) (2, 3). As of May 10, 2020, there are more than 3,917,366 confirmed cases of COVID-19 and 274,361 deaths worldwide (4). The clinical spectrum of COVID-19 infection appears to be wide. In addition to the typical symptoms of severe viral pneumonia, critically ill patients also suffer from acute kidney injury, acute cardiac injury, as well as multiple organ failure (5, 6). It has been known that COVID-19 invades respiratory epithelial cells *via* the receptor angiotensin converting enzyme II (ACE2) (7, 8). However, ACE2 expression is not limited to the lung, and extrapulmonary expression of ACE2 is also found in heart, vessels, kidney and digestive systems, which may contribute to the non-respiratory symptoms observed in NCIP patients (9).

The epidemiological and clinical characteristics of COVID-19 have been widely reported recently (10). Sepsis is the most frequently observed complication in patients with COVID-19 (11, 12). Notably, another common complication of severe COVID-19 illness, shock, is observed in 23~31% of COVID-19 patients in ICU (5, 13). Moreover, in a recent retrospective cohort study, septic shock was observed in 70% of non-survival patients with COVID-19 (38 of 54 patients) (6).

Prolonged critical illness is associated with adrenocortical dysfunction (14). The term “critical illness-related corticosteroid insufficiency” (CIRCI) is defined as aberrant synthesis and secretion of cortisol, and cellular corticosteroid activity that is inadequate for the severity of the patient’s critical illness (15). CIRCI is thought to occur in several critical conditions, including sepsis and septic shock, severe pneumonia and acute respiratory distress syndrome (ARDS) (15). CIRCI is associated with an increase in circulating levels of biological markers of inflammation and coagulation over time (16), which is seen frequently in patients with severe COVID-19. However, it remains unknown whether adrenocortical dysfunction occurs in COVID-19 patients.

The present study summarized the clinical features of COVID-19 reported in currently available observational studies, and examined ACE2 expression in human adrenal glands. Serum cortisol levels were measured in critically ill patients with or without COVID-19. The main goal of this study was to provide theoretical rationale for the use of corticosteroids in critical ill patients with COVID-19.

## Materials and Methods

### Reviewing the Reports of Vasopressors Use in COVID-19 Critically Ill Patients

We searched for clinical studies on critically ill patients with SARS-COV-2 infection published from January 1^st^, 2020 to May 1^st^, 2020. The searched databases included “pubmed”, “Scopus” and “Web of Sciences”. The search terms included: “Novel coronavirus”, “Coronavirus disease”, “COVID-19”, “2019-nCoV”, “SARS-COV-2”, “Wuhan coronavirus”, and “Wuhan pneumonia”. Only clinical observational research studies and clinical studies were included. The language was limited to English.

The inclusion criteria were as follows: (1) age ≥18 years old; (2) SARS-CoV-2 in respiratory tract specimens, detected by quantitative reverse-transcription PCR (q-RT-PCR); (3) PaO_2_/FiO_2_ ≤300 mmHg; (4) presence of least one subgroup reporting critically ill patients receiving vasopressors. The exclusion criteria were as follows: (1) neuroendocrine system diseases (including disorders of hypothalamus, pituitary gland, or adrenal gland); (2) long-term hormone use; (3) mental diseases; (4) pregnancy. The retrieval of the data was done independently by two researchers. Disagreements were resolved by discussion with the third researcher. The following patient information was recorded: number of patients, the male to female ratio, acute physiology and chronic health evaluation II (APACHE II) score, sepsis-related organ failure assessment (SOFA) score, number of patients with cardiovascular disease, endocrine disease, cardiovascular complications due to SARS-CoV-2 infection, including shock, myocardial injury, arrhythmia, use of vasopressors or extracorporeal membrane oxygenation (ECMO) support, and mortality.

### Ethics and Clinical Registration

The study was reviewed and approved by the Ethics Committee of Xinhua Hospital Affiliated to Shanghai Jiaotong University, School of Medicine and Shanghai Public Health Center (Number XHEC-D-2020-073). The Ethics Committee evaluated the design and implementation process of the study. The researchers strictly followed the medical ethics guidelines of the “Helsinki Declaration” and “International Ethical Guidelines for Human Health Related Research”. All the patients had signed the “Extensive Informed Consent” when they were admitted to the hospital. The study was registered at the Chinese Clinical Trial Registry (http://www.chictr.org.cn) with registration number ChiCTR-ORC-16008623.

### Immunohistochemical and Immunofluorescent Analysis

Healthy tissue specimens around tumors surgically resected from inpatients in Xinhua Hospital affiliated to Shanghai Jiaotong University School of Medicine from Jan 2020 to Apr 2020, were collected. Specimens included three samples of lung tissues and the same number of small intestines tissues, thyroid tissues, adrenal tissues, and adrenal glands surgically removed from pheochromocytoma patients for histological examination and immunohistochemical analysis.

The tissues were then fixed in 4% paraformaldehyde, and processed for standard (4-mm) paraffin sectioning. Fully automated immunohistochemistry (IHC) stainer (LEICA Bond RX) was used for the staining. Briefly, sections were incubated with primary antibodies against ACE2(Item No.:ab108209, Abcam, USA)at a dilution of 1: 100 or transmembrane protease serine 2 (TMPRSS2) at a dilution of 1:1,000 for 30 min and the bound antibodies were detected by DAB (3, 3’-diaminoben-zidine) staining system. The same concentration of normal IgG served as a negative control. In the adrenal tissue sections, hematoxylin-eosin (H&E) staining was also performed for histological localization. Co-staining of anti-ACE2 and anti-TMPRSS2 or anti-CYP11B1 (Item No.:MABS502, Sigma-Aldrich, USA) antibodies at a dilution of 1:100 was performed for immunofluorescence analysis.

### Retrospective Analysis of Serum Cortisol Level in COVID-19 and Non-COVID-19 Critically Ill Patients

This study included COVID-19 critically ill patients in the intensive care unit (ICU) of the Shanghai Public Health Center from March 1st to May 1st, 2020. The inclusion criteria were as follows: (1) age ≥18 years old; (2) SARS-CoV-2 positive result from respiratory tract specimens as confirmed by quantitative reverse-transcription PCR (q-RT-PCR) detection; (3) PaO2/FiO2 ≤200 mmHg; (4) total plasma cortisol concentration measured in ICU. Exclusion criteria for the study were as follows: neuroendocrine system diseases (including disorders of hypothalamus, pituitary gland or adrenal gland), long-term hormone use (more than 7 days), basic pulmonary diseases, mental diseases, and pregnancy.

The control group of non-COVID-19 critically ill patients came from the ICU of Xinhua Hospital, affiliated to Shanghai Jiaotong University, School of Medicine. The inclusion and exclusion criteria were consistent with the group of COVID-19 critically ill patients. The total plasma cortisol was measured at 8am by the assay from Beijing North Institute of Biotechnology Co., Ltd and its reference range was 6.7-22.6 ug/fl. We selected the first measure of the total plasma cortisol for data analysis. We collected all the basic information and factors affecting serum cortisol level, including age, sex, APACHE II score, diagnosed cardiovascular and endocrine system diseases, use of hormones, administration of etomidate or azole antifungals during treatment, use of vasopressors as well as ventilators or ECMO support.

### Statistical Methods

SPSS 26.0 software was used to analyze all the data in this study. Continuous variables with normal distribution were expressed as Mean ± SD, while non-normally distributed data were expressed as median ± interquartile range. For data comparison between COVID-19 group and non- COVID-19 group, Student’s t test or Mann Whitney U test was used to evaluate continuous variables. Chi-square test or Fisher’s exact test was used to assess categorical variables. P values ≤ 0.05 was considered statistically significant.

## Results

### Critically Ill Patients With SARS-COV-2 Infection Have a High Incidence of Vasopressors Use

A total of 438 studies were initially selected based on the search strategy. After screening the title and abstract, 54 articles were selected for a full text evaluation. Of them, 26 were excluded due to lack of molecular diagnostic information (3), age of the patients (<18) (17) or pregnancy status (7). 23 additional articles were excluded due to lack of reports of vasopressors or shock. Finally, five clinical studies on critically ill patients with SARS-COV-2 infection were analyzed (5, 13, 17–19) (**Supplement Figure 1**). **Table 1** shows the main characteristics of the included studies and the cardiovascular complications of patients during treatment.

**Table 1:** Cardiovascular complications in patients with SARS-CoV-2 infections.

Cohort	Cao et al.	Wang et al.	Arentz et al.	Huang et al.	Yang et al.
No. of patients	199	36	21	13	52
Age, years	58.0 (49.0–68.0)	66 (57–78)	70(43–92)	49.0(41–61)	59.7-13.3
Sex ratio (F:M)	79:120	14:22	10:11	2:11	17:35
APACHE II	**-**	17 (10–22)	**-**	**-**	17(14-19)
SOFA	**-**	5 (3–6)	**-**	**-**	**-**
Cardiovascular disease	**-**	21 (58%)	9 (43%)	3 (13%)	5 (10%)
Hypertension	**-**	9 (25%)	**-**	2 (15%)	**-**
Endocrine disease	23 (12%)	8 (22%)	7 (33%)	1(8%)	9 (17%)
Cardiovascular complications					
Use of vasopressors	44 (22%)	**-**	14(67%)		18 (35%)
Shock	4 (2%)	11 (31%)	**-**	3 (23%)	**-**
Myocardial injury	**-**	8 (22%)	7 (33%)	4 (31%)	12 (23%)
Arrhythmia	1(0.5%)	16 (44%)	**-**	**-**	**-**
Acute heart failure	1(0.5%)	**-**	4(19.1)	**-**	**-**
Use of ECMO	4 (2%)	4 (11%)	**-**	2 (15%)	6 (12%)
Mortality	44 (22%)	11 (31%)	11(52%)	6 (46%)	32 (62%)

Of these 5 clinical studies on critically ill COVID-19 patients, 2 articles reported both critically ill patients and mildly ill patients, and the patients were subgrouped according to the severity of their condition (5, 13). We selected the critically ill patients’ data. Three articles reported that 22%–67% of COVID-19 patients used vasopressors (17–19).

Nonetheless, the incidence of hypotension may still be underestimated. One possible reason is that many patients were still in hospital at the end of the included studies, thus still potentially at risk of developing hypotension. Cao et al. reported that the proportion of patients receiving vasopressor therapy was much higher than the proportion of patients with shock (17). Overall, the incidence of shock was between 4-31%, and lower than the incidence of patients that received vasopressor therapy. In addition, the incidence of myocardial injury patients was between 22%–33%, and the mortality rate was between 22%–62%.

### Immunohistochemical and Immunofluorescent Analysis

**Figure 1:**
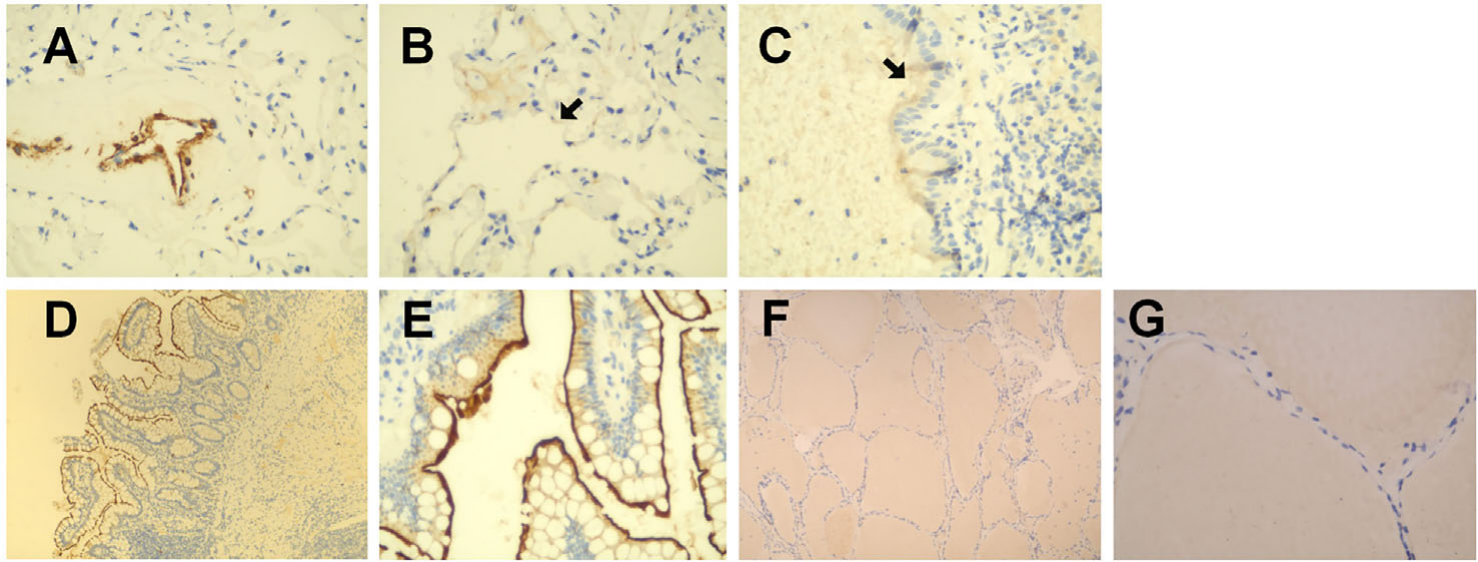
Immunohistochemical staining of ACE2 in human healthy lung tissues. small intestines and thyroid tissues. **(A–C)** in healthy lung tissue sections, ACE2 was widely expressed in pulmonary vascular endothelial cells, alveolar epithelial cells, bronchial mucosal epithelial cells, and on the apical surface of ciliated columnar epithelial cells. **(D, E)** in human normal small intestine sections, ACE2 was mainly expressed on the surface of villi epithelial cells. **(F, G)** ACE2 immunoreactivity was not observed in human normal thyroid tissue sections. Original magnification: **(A–C, E, G)**: ×400; D&F:×100.

As shown in **Figures 1A–C**, in healthy lung tissue sections ACE2 was widely expressed in pulmonary vascular endothelial cells, alveolar epithelial cells, bronchial mucosal epithelial cells, and on the apical surface of ciliated columnar epithelial cells. In human normal small intestine sections, ACE2 was mainly expressed on the surface of villi epithelial cells (**Figures 1D, E**). However, ACE2 immunoreactivity was not observed in normal human thyroid tissue sections (**Figures 1F, G**).

**Figure 2:**
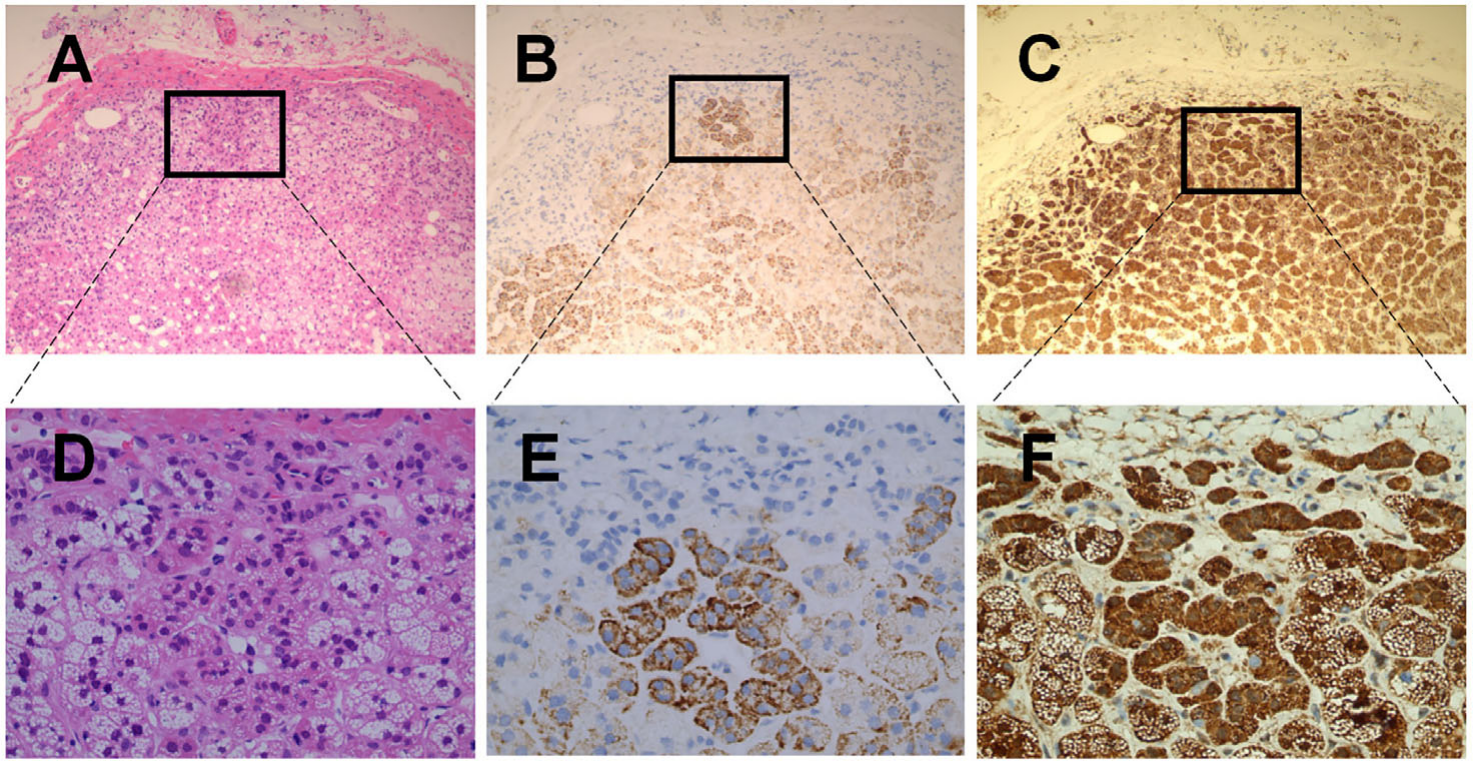
Haematoxilin-Eosin staining and immunohistochemical staining of ACE2 & TMPSS2 in human healthy adrenal tissues. **(A–C)** were the serial sections of the same adrenal cortex. Panel A showed Haematoxilin-Eosin (HE) staining. **(B, C)** were stained with antibodies aganist ACE2 **(B)** and TMPSS2 **(C)**, respectively. Original magnification: ×100. Areas in black boxes in **(A–C)** were shown enlarged in **(D–F)** (×400), respectively.

As shown in the **Figures 2B, E**, immunohistochemical staining of ACE2 in human adrenal gland sections showed no obvious immunoreactivity in the zona glomerulosa, which is the unique source of the mineralocorticoid aldosterone (19). In contrast, ACE2 was widely distributed in the zona fasciculata/reticularis, which produces the glucocorticoids and the androgens. Transmembrane Serine Protease 2 (TMPRSS2) appears to prime the viral spike (S) protein to enhance ACE2-mediated SARS-CoV-2 entry (7). As shown in **Figures 2C, F**, we found that TMPRSS2 was widely expressed in all three zones of the adrenal cortex. **Figures 2A, D** showed the observation place with HE staining.

**Figure 3:**
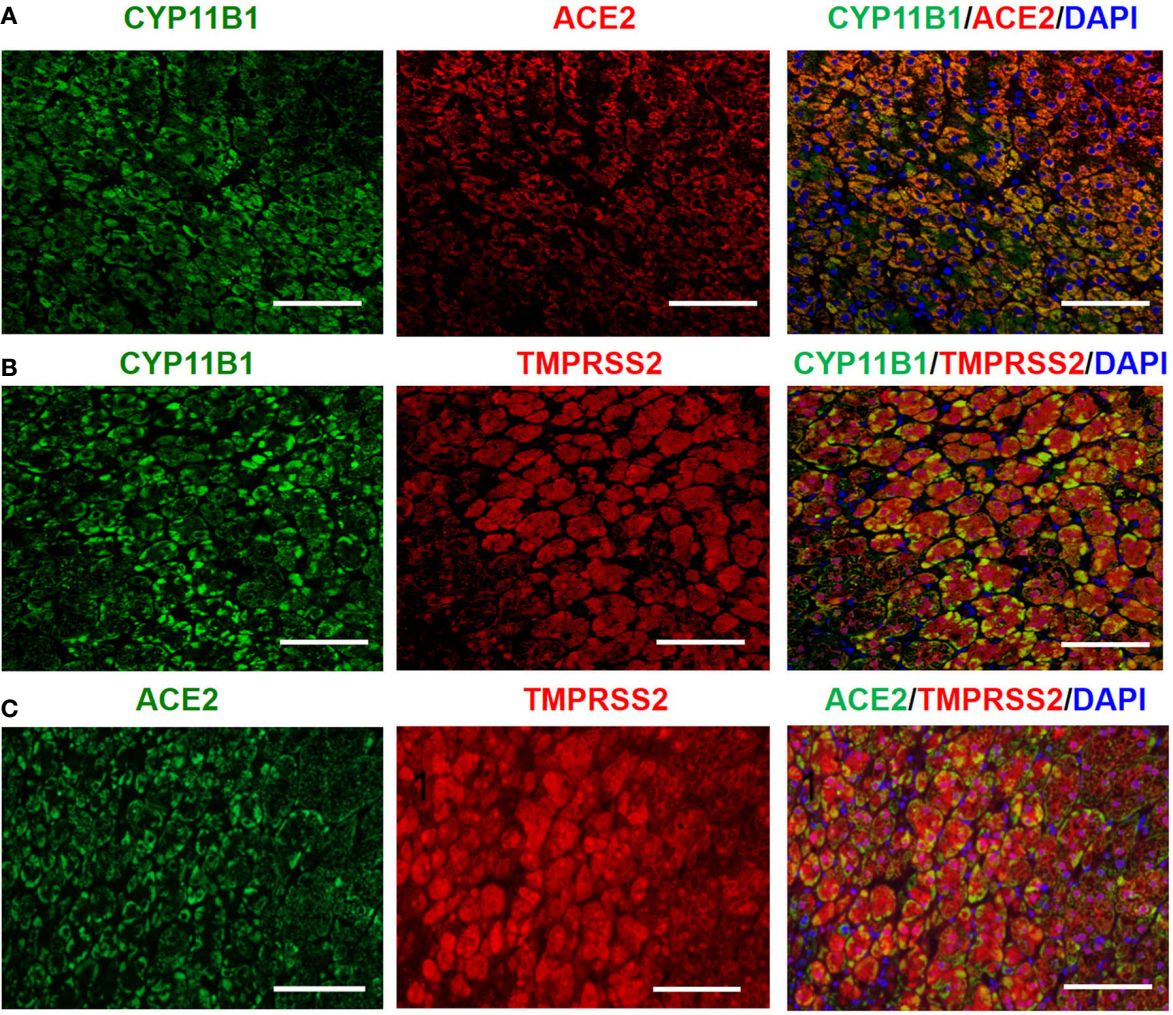
Double immunofluorescence staining demonstrates the colocalization of CYP11B1/ACE2 **(A)**, CYP11B1/TMPRSS2 **(B)** and ACE2/TMPRSS2 **(C)** in human healthy adrenal tissues. Original magnification: ×200. Scale bars correspond to 50 μm.

Steroid 11-hydroxylase (CYP11B1), an enzyme catalyzing the terminal steps of cortisol synthesis, is mainly expressed in the human adrenal zona fasciculata/reticularis, and determines the functional differentiation of adrenocortical cells (20). As shown in **Figure 3**, double immunofluorescence staining clearly demonstrated the colocalization of CYP11B1/ACE2, CYP11B1/TMPRSS2 and ACE2 /TMPRSS2 in the adrenal cortex.

**Figure 4:**
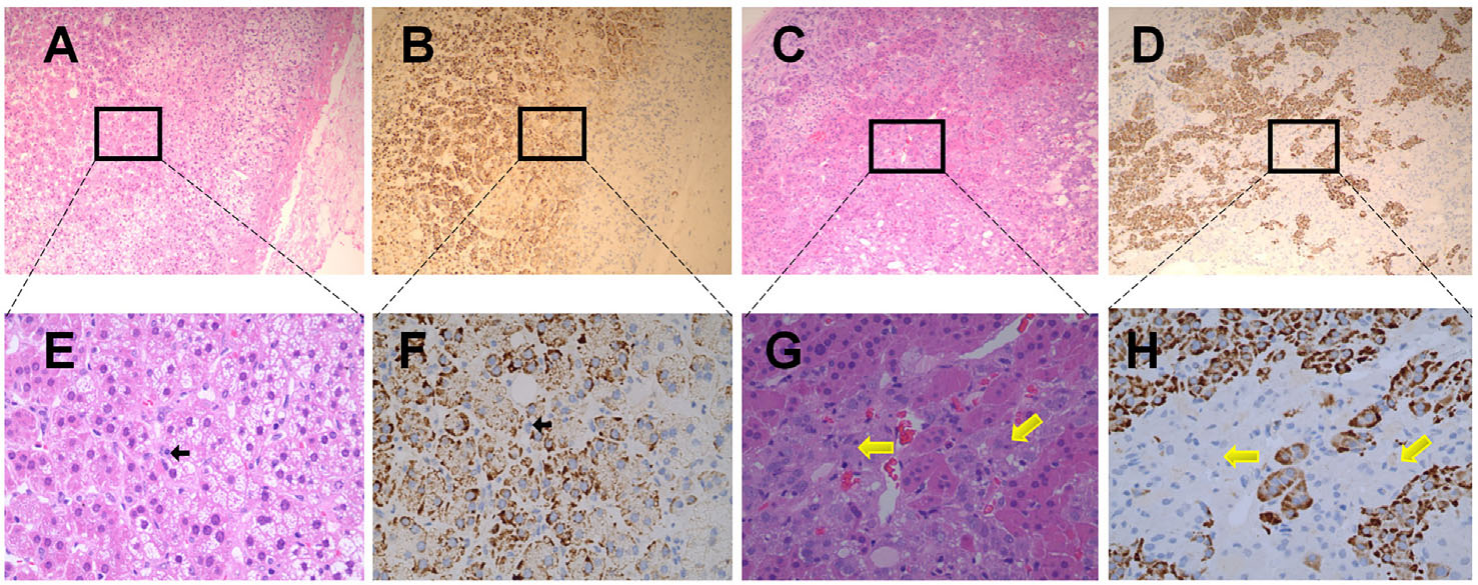
Immunohistochemical staining of ACE2 in human normal adrenal medulla and pheochromocytoma tissues. **(A, B)** were the serial sections of the same adrenal medulla. **(C, D)** were the serial sections of the same pheochromocytoma tissues. **(A, C)** showed Haematoxilin-Eosin (HE) staining. **(B, D)** were stained with antibodies aganist ACE2. Original magnification: ×100. Areas in black boxes in **(A–D)** were shown enlarged in **(E–H)** (×400), respectively. In the healthy adrenal tissues, c cells (pointed by black arrows) could only be distinguished in the adrenal medulla under high magnification **(E, F)**, and exhibited no obvious ACE2 immunoreactivity. In sections obtained from pheochromocytoma tissues, ACE2 immunoreactivity was also not observed in pheochromocytoma cells (pointed by yellow arrows).

The expression of ACE2 was also detected in the adrenal medulla. In the healthy adrenal tissues (**Figures 4B, F**), chromaffin cells (indicated by black arrows) could only be distinguished in the adrenal medulla under high magnification (×400), and exhibited no obvious ACE2 immunoreactivity. To further confirm these findings, sections obtained from pheochromocytoma tissues were stained with ACE2 antibody. As shown in **Figures 4D, H**, ACE2 immunoreactivity was not observed in pheochromocytoma cells (indicated by yellow arrows). **Figures 4A, E** showed the observation place in adrenal medulla. **Figures 4C, G** showed the observation place in pheochromocytoma tissues.

### The Cortisol Levels of Critically Ill Patients With COVID-19 Are Lower Than Those in Non-COVID-19 Critically Ill Patients

Since the epidemic continued to decline in China, there were only nine critically ill patients with COVID-19 in the ICU of Shanghai Public Health Center by the end of this study. All the nine patients met the inclusion criteria without exclusion criteria, and were recruited in this study.

**Figure 5:**
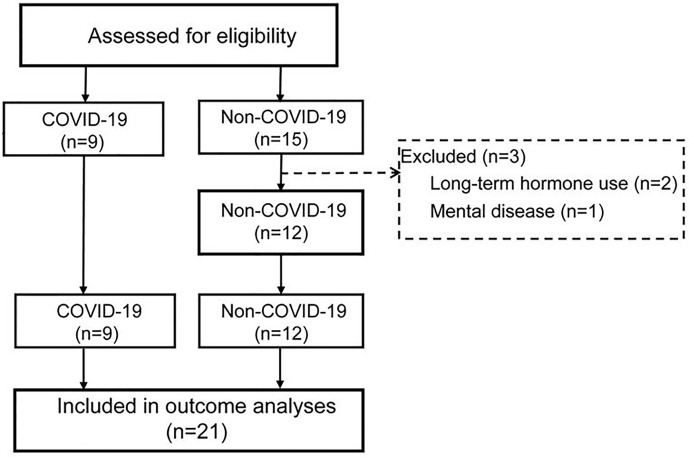
Flowchart of trial procedures. All the COVID-19 patients met the inclusion criteria: 1. Age ≥18 years, 2. Positive results of sars-cov-2 by qRT-PCR, 3. PaO2/FiO2 ≤ 200mmHg, 4. Total plasma cortisol was measured. Patients did not receive prolonged hormonal therapy, and do not have hypothalamus, pituitary gland, adrenal gland disease or mental illness; Female patients were not pregnant.

Of the 15 non-COVID-19 critically ill patients, 3 patients did not meet the inclusion criteria and were excluded. In the end, nine COVID-19 and 12 non-COVID-19 patients were included in this study (**Figure 5**). During treatment, 88.9% of COVID-19 critically ill patients used vasopressors, while 100% non-COVID-19 critically ill patients used vasopressors. Thus, there was no significant difference between the two groups (P = 0.429). 88.9% of COVID-19 critically ill patients and 100% of non-COVID-19 critically ill patients needed ventilator support (P = 0.429). 77.7% of COVID-19 patients received Vein-Artery ECMO support, and none of non-COVID-19 patients required ECMO support (P = 0.000).

**Figure 6:**
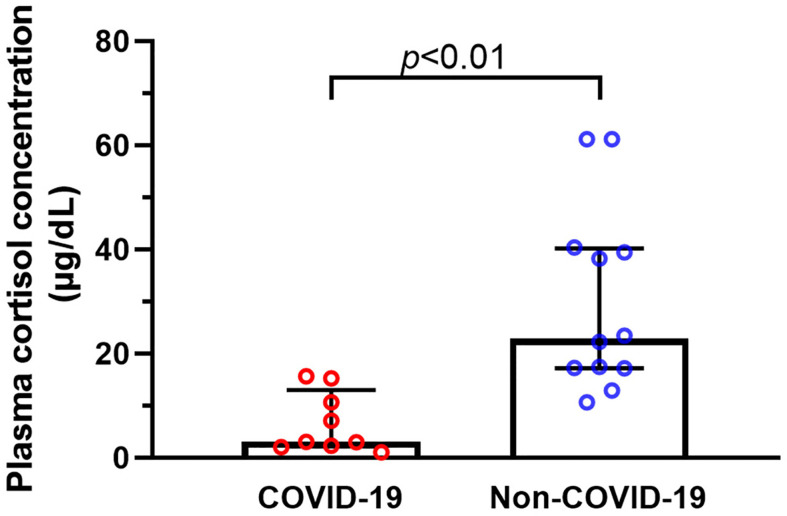
Plasma cortisol concentrations of critically ill patients with or without COVID-19. We collected data on plasma cortisol concentrations in nine critically ill patients with COVID-19, and the results showed that the cortisol levels of COVID-19 critically ill patients were considerably lower than those in non-COVID-19 critically ill patients.

We compared the differences in plasma cortisol levels between the two groups. Concentrations of cortisol in COVID-19 patients were considerably lower than those in non-COVID-19 critically ill patients (P = 0.000) (**Figure 6**). It was worth noting that random plasma cortisol elevated beyond the normal range in 50% non-COVID-19 critically ill patients. In contrast, six of the nine COVID-19 critically ill patients had plasma cortisol concentrations below 10 µg/dl, which met the criteria for the diagnosis of CIRCI (21). Plasma cortisol levels are known to be affected by several factors, such as use of etomidate, glucocorticoids or azole antifungals, and bile acids level in blood (22). Therefore, we evaluated the differences in these factors between the two groups (**Table 2**). We did not detect significant difference in the composition ratio of using glucocorticoids (P = 0.063), or using the azole antifungals (P = 0.063) between the two groups. The level of bile acid was similar between the two groups (P = 0.310). None of the patients in the two groups used etomidate.

**Table 2:** Clinical characteristics of the critically ill patients and complication in ICU.

Characteristic	COVID-19 group (n=9)	Non-COVID-19 group (n=12)	*p*
Age, mean (SD), year	71.7 (8.1)	68.9(20.2)	0.706
Sex ratio (F:M)	8:1	8:4	0.338
APACHE II, mean (SD)	25.4(2.3)	24.3(2.6)	0.330
**Coexisting conditions, no.**			
Cardiovascular disease	1/9	2/12	1
Endocrine disease	1/9	1/12	1
**Treatment in ICU**			
Glucocorticoid(in 7 day)	3/9	0/12	0.063
Etomidate	0/9	0/12	>0.05
Azole antifungals	3/9	0/12	0.063
**Complication in ICU**			
Bile acid (umol/l), median (IQR)	5.96 (4.10-9.75)	4.2 (1.82-10.35)	0.345
Use of vasopressors	8/9	12/12	0.429
Use of ventilator	8/9	12/12	0.429
Use of V-A ECMO	7/9	0/12	0.000

## Discussion

To date, the ongoing COVID-19 pandemic has affected more than 210 countries and territories around the world (1). The epidemiological and clinical features of COVID-19 have been widely reported (2, 3, 5, 6). However, mechanisms underlying the pathogenesis of COVID-19 still need to be fully elucidated. Previous studies have identified the main path for SARS-CoV-2 entry into the cell – namely *via* the viral spike (S) protein attaching to ACE2 and employing the cellular serine protease (TMPRSS2) for S protein priming in human lung cells (7, 23). Thus, COVID-19 patients display characteristic respiratory symptoms including dyspnea, low oxygen saturation, and rapid progress of chest radiological abnormality (24). In addition, several studies have provided bioinformatic evidence of ACE2 expression in cardiovascular, digestive, urinary, and reproductive systems, implying that these organs are all potential routes of SARS-CoV-2 infection (9, 25, 26). Consistent with these studies, our data confirmed that ACE2 protein expression was enriched in pulmonary epithelial/endothelial cells and the enterocytes of small intestine tissues.

Based on the systematic literature review (17–19), we found that 22%~67% of patients infected with SARS-CoV-2 received vasopressors therapy, due to septic shock induced by SARS-CoV-2 or secondary infections including bacteria and fungi. On the other hand, given that SARS-CoV-2 pandemic seems to be particularly deleterious to patients with underlying cardiovascular diseases (CVD), including congestive heart failure (27), vasopressors may also be used in patients with cardiogenic shock. Notably, a recent bioinformatic study indicates medium-level expression of ACE2 in adrenal glands (28). The hormones produced by the adrenal cortex and adrenal medulla, specifically steroid hormones and catecholamines, play critical roles in the regulation of vascular reactivity (29, 30). We investigated the presence and localization of ACE2 protein in human adrenal glands by immunohistochemistry. ACE2 immunostaining was not observed in adrenal medulla obtained from normal adrenal glands or from adrenal glands with pheochromocytoma. However, both ACE2-positive and TMPRSS2-positive immunostained cells were widely observed in zona fasciculata and zona reticularis of adrenal cortex that are mainly responsible for synthesis and secretion of glucocorticoids. This finding was further confirmed by the colocalization of ACE2 or TMPRSS2 with CYP11B1, a marker for the functional differentiation of cells in the zona fasciculata and reticularis. These results suggest that SARS-CoV-2 may potentially directly target zona fasciculata/reticularis of adrenal cortex, thereby influencing circulating glucocorticoid levels.

The importance of glucocorticoids has been illustrated in studies that demonstrated the crucial role of the adrenal glands for survival under conditions of stress (31, 32). Glucocorticoids have profound metabolic, cardiovascular and immunological roles in adequate stress response that allows to maintain and restore homeostasis in the human body (33). Previous studies showed that elevated plasma concentrations of cortisol in critically ill patients are evoked by the activation of the HPA axis and systemic hyperinflammation (34). Consistent with this notion, we found that random plasma cortisol levels were elevated beyond the normal range in 50% of non-COVID-19 critically ill patients. On the other hand, random plasma cortisol levels in COVID-19 critically ill patients were considerably lower than those in non-COVID-19 ones. It’s worth noting that 67% of critically ill patients with COVID-19 had random plasma cortisol concentrations below 10 µg/dl, which met the criteria for the diagnosis of CIRCI (21). By mediating and enhancing the action of angiotensin II and catecholamines, cortisol maintains cardiac contractility, vascular tone, and blood pressure, which are crucial for critically ill patients (35). Therefore, the cardiovascular symptoms of CIRCI include hypotension refractory to fluid resuscitation and decreased sensitivity to catecholamines (21). This was also supported by our findings obtained from the systematic literature review, which showed that vasopressors were required in 22%~67% of COVID-19 patients.

Previous studies have identified dysregulation of the HPA axis and impaired steroidogenesis as major pathophysiologic events that account for the development of CIRCI (21). For example, circulating bile acids can pass the blood–brain barrier and bind to the hypothalamic glucocorticoid receptor, causing central HPA suppression, and consequent decrease in ACTH release and adrenocortical steroidogenesis (36). Another possible cause of ICU-acquired CIRCI is the administration of drugs that interfere with steroidogenesis, such as etomidate, azole antifungals, and exogenous glucocorticoid therapy (37). In this study, we found no significant difference in circulating levels of bile acids, utilization of etomidate, azole antifungals, and glucocorticoids between non-COVID-19 and COVID-19 critically ill patients. Our results suggest therefore that these factors do not contribute to the considerably lower cortisol levels in COVID-19 critically ill patients. Components of various pathogens, including bacteria and viruses, have been found to directly impact adrenocortical steroidogenesis (38–40). The present study found the colocalization of ACE2 and TMPRSS2 in the zona fasciculata and zona reticularis of adrenal cortex. Our findings suggest that adrenocortical cells in zona fasciculata and zona reticularis may be potential targets of SARS-CoV-2 infection, thereby providing a possible cause for the reduction of cortisol levels in COVID-19 critical ill patients. Just on June 16, 2020, a study published in “Nature” found that a cheap, widely available steroid drug- dexamethasone can reduce COVID-19 deaths by up to one third in critically ill patients. However, the steroid had no effect on people with mild cases of COVID-19 (41). The authors speculate that the therapeutic effect of dexamethasone on critically ill COVID-19 patients may be related to its anti-inflammatory response. However, it should be noted that most critically ill patients may have CIRCI, and the therapeutic effect of dexamethasone may also be related to its improvement of cortical function. Our research was focused on the latter and could partly explain why dexamethasone treatment reduced mortality in severely ill patients with COVID-19.

The reproductive implications of coronavirus infection recently have attracted much attention. Several studies clearly demonstrated that ACE2 is highly expressed in spermatogonia, leydig and sertoli cells, as well as in cells of the seminiferous tubules in the human testis (42–44). Thus, the binding of the virus to these ACE2-positive cells may cause severe alteration of testicular tissue, and therefore have a serious impact on fertility. In addition, the effect of SARS-CoV-2 infection on male sex hormones has recently been investigated in a single center-based study (45). In this study, Ma et al. found that serum luteinizing hormone (LH) was significantly increased, but the ratio of testosterone (T) to LH and the ratio of follicle stimulating hormone (FSH) to LH were dramatically decreased in reproductive-aged males with COVID-19 compared to age-matched healthy men. The zona fasciculata and zona reticularis of adrenal cortex are recognized as the main source of extragonadal production of androgens (dehydroepiandrosterone [DHEA] and DHEA sulfate [DHEAS]) (46). The adrenal androgens are converted into androstenedione and then into testosterone in the peripheral tissues. The importance of the adrenal-derived androgens to the overall production of sex steroid hormones is highlighted by the fact that approximately 50% of total androgens in the prostate of adult men are derived from adrenal steroid precursors (19). In the present study, we found that both ACE2 and TMPRSS2 proteins were expressed in the zona fasciculata/reticularis of the human adrenal cortex. These results may indicate that SARS-CoV-2 infection might impact extragonadal androgen production by targeting adrenocortical steroidogenic cells.

Our study has several limitations. First, the results of this study need to be interpreted with caution because of its retrospective nature and the small sample size. Retrospective study with small sample size always increases the likelihood of a Type II error and decreases statistical power. In the retrospective study, there is no way to control when and how often the total cortisol measured, which would also result in some degree of research bias. Second, this study used the APACHE II score to evaluate the severity of the patients’ illness. Notably, seven of the nine COVID-19 critically ill patients received ECMO therapy to improve gas exchange. Since several important parameters related to gas exchange were included in the APACHE II scoring system, the severity of disease might be underestimated in COVID-19 critically ill patients requiring ECMO support. Third, vasopressors were used in 20 of the 21 critically ill patients recruited in this study, thereby making it difficult to assess the relationship between plasma cortisol level and haemodynamic instability. Although intravenous glucocorticoids are commonly used in patients with severe SARS or MERS pneumonia, lack of their effect on the overall survival made their use to treat SARS-CoV-2 infection questionable (47). WHO guidance on management of COVID-19 advises against corticosteroids, unless indicated for other reasons, such as adrenal insufficiency (47). A growing body of evidence reveals that dysfunction of the HPA axis occurs frequently in patients with serious infections (48, 49). The colocalization of ACE2 and TMPRSS2 in zona fasciculata and zona reticularis of adrenal cortex suggests that SARS-CoV-2 may directly interfere with the process of cortisol synthesis, thereby further increasing the incidence of CIRCI in COVID-19 critical ill patients.

Our findings suggest that the adrenal cortex may be the target organ of SARS-CoV-2. In comparison with non-COVID-19 critically ill patients, a lower random plasma cortisol level was observed in COVID-19 critically ill patients, among whom 67% met the criteria for the diagnosis of CIRCI. Since CIRCI is characterized by hypotension unresponsive to fluid resuscitation, and requires vasopressor therapy, it is necessary to carefully consider whether to add an appropriate dose of corticosteroids to maintain the stability of the circulatory system. Our findings recommend measuring plasma cortisol levels to guide hormonal therapy.

## Data Availability Statement

The raw data supporting the conclusions of this article will be made available by the authors, without undue reservation.

## Ethics Statement

The studies involving human participants were reviewed and approved by Ethics Committee of Xinhua Hospital Affiliated to Shanghai Jiaotong University, School of Medicine and Shanghai Public Health Center (Number XHEC-D-2020-073). The patients/participants provided their written informed consent to participate in this study.

## Author Contributions

LJ, YG, and XZ conceived and performed experiments and wrote the manuscript. YM, BX, and WG performed experiments. FL and RR provided data analysis. All authors contributed to the article and approved the submitted version.

## Conflict of Interest

The authors declare that the research was conducted in the absence of any commercial or financial relationships that could be construed as a potential conflict of interest.

## Supplementary Material

Supplementary Figure 1 The PRISMA diagram of literature review.
