# Supplementary figures and images for: The Adrenal Cortex, an Underestimated Site of SARS-CoV-2 Infection
Source: Front Endocrinol (Lausanne). 2021 Jan 8;11:593179. doi: 10.3389/fendo.2020.593179 (PMC7820749; doi:10.3389/fendo.2020.593179)

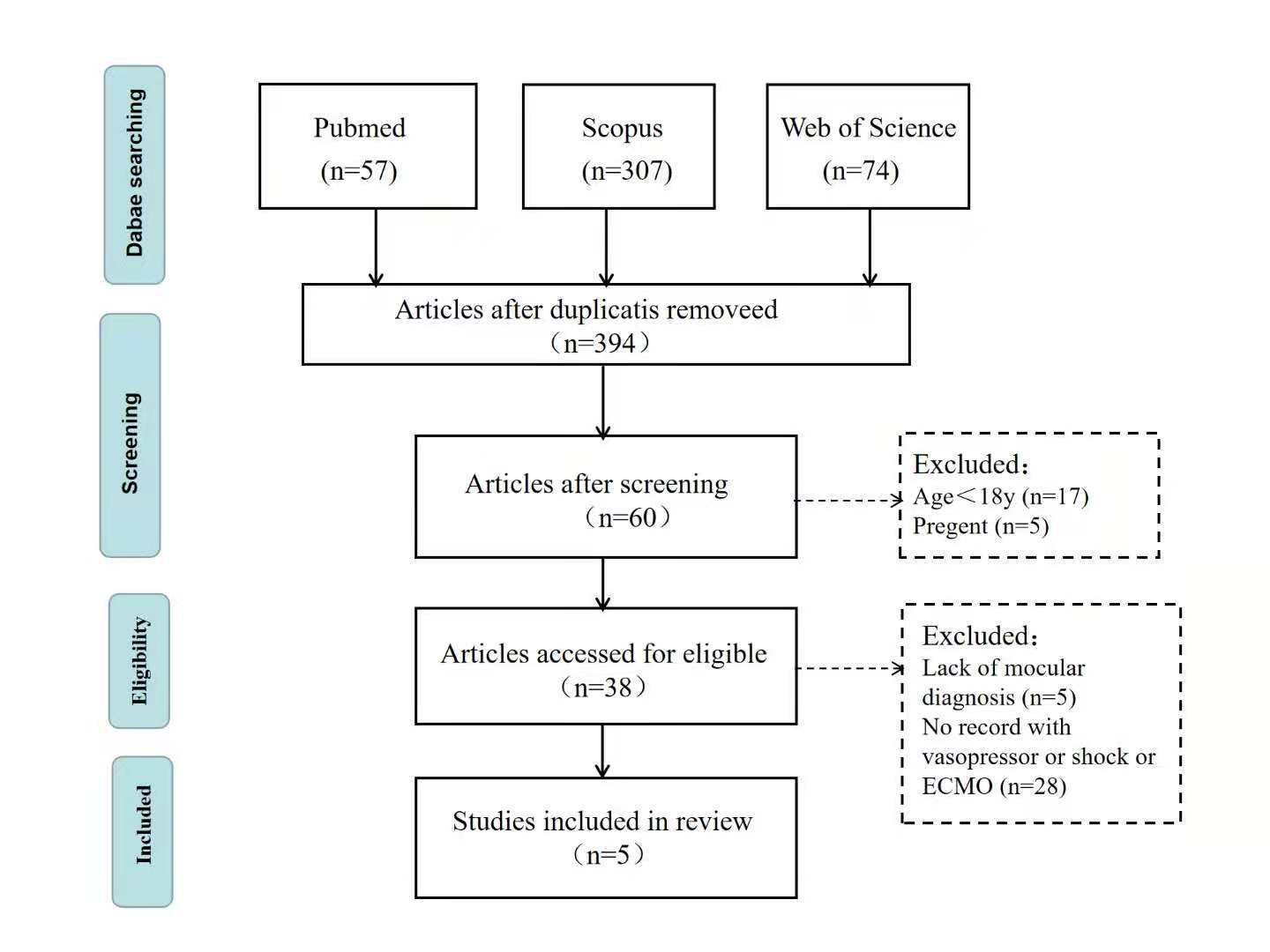

Supplement: Supplementary Figure 1 — The PRISMA diagram of literature review. [file Image_1.jpeg]
